# A Case of Tumor-Induced Osteomalacia Masked by Parathyroid Carcinoma

**DOI:** 10.3390/jcm15114368

**Published:** 2026-06-05

**Authors:** Giulia Manfredi, Anna Turisani, Alberto Piasentier, Chiara Dobrinja, Mattia Mario, Chiara Ratti, Luigi Murena, Bruno Fabris, Veronica Calabro’, Stella Bernardi

**Affiliations:** 1 Department of Medical, Surgical and Health Sciences, University of Trieste, Cattinara University Hospital, Strada di Fiume 447, 34149 Trieste, Italy; giulia.manfredi@studenti.units.it (G.M.); anna.turisani@studenti.units.it (A.T.); mattia.mario@asugi.sanita.fvg.it (M.M.); lmurena@units.it (L.M.); b.fabris@fmc.units.it (B.F.); 2 Department of Biomedical Sciences, Humanitas Research University, Via Rita Levi Montalcini 4, 20072 Pieve Emanuele (MI), Italy; alberto.piasentier@humanitas.it; 3 General Surgery Unit, Azienda Sanitaria Universitaria Giuliano Isontina (ASUGI), Cattinara University Hospital, Strada di Fiume 447, 34149 Trieste, Italy; chiara.dobrinja@units.it; 4 Pathology Unit, Azienda Sanitaria Universitaria Giuliano Isontina (ASUGI), Cattinara University Hospital, Strada di Fiume 447, 34149 Trieste, Italy; 5 Orthopaedics and Traumatology Unit, Azienda Sanitaria Universitaria Giuliano Isontina (ASUGI), Cattinara University Hospital, Strada di Fiume 447, 34149 Trieste, Italy; chiara.ratti@asugi.sanita.fvg.it; 6 Endocrinology Unit, UCO Medicina Clinica, ASUGI, Cattinara University Hospital, Strada di Fiume 447, 34149 Trieste, Italy; veronica.calabro@asugi.sanita.fvg.it

**Keywords:** hypophosphatemia, tumor-induced osteomalacia, TIO, hyperparathyroidism, parathyroid adenoma, parathyroid carcinoma, granulomatous giant-cell reaction, case report

## Abstract

**Background:** Tumor-induced osteomalacia (TIO) is a rare paraneoplastic syndrome caused by fibroblast growth factor 23 (FGF-23)-secreting tumors, typically of mesenchymal origin, leading to renal phosphate wasting and severe bone demineralization and fragility fractures. Diagnosing TIO remains a significant clinical challenge, particularly when coexisting mineral metabolism disorders, such as hypercalcemic hyperparathyroidism, are masking its clinical presentation. **Case Presentation:** A 74-year-old woman with fragility fractures, generalized bone pain, and nephrolithiasis was initially diagnosed with primary hyperparathyroidism due to concomitant hypercalcemia, hypophosphatemia, and elevated parathyroid hormone (PTH). Despite a successful parathyroidectomy, which normalized calcium levels, severe hypophosphatemia persisted due to renal phosphate wasting. High FGF-23 levels and subsequent functional imaging indicating a somatostatin receptor-positive lesion in the left popliteal fossa led to the diagnosis of TIO. Surgical resection immediately normalized FGF-23 levels, leading to a slower rise in phosphorus during follow-up. Histopathology revealed a tophaceous-like giant cell granulomatous reaction, recalling the earlier report by Prader. **Conclusions:** This case highlights that parathyroid disorders can coexist with TIO, and they may delay its diagnosis. In this circumstance, a high index of clinical suspicion is represented by the persistence of hypophosphatemia post-parathyroidectomy.

## 1. Introduction

Tumor-induced osteomalacia (TIO) is a rare paraneoplastic syndrome caused, in most cases, by benign and small phosphaturic mesenchymal tumors (PMTs) secreting fibroblast growth factor 23 (FGF-23), which are preferentially located in the soft tissues of the extremities, long bones and the nasopharynx [1,2]. In addition, FGF-23 excess has been documented also in association with various malignancies [3]. It is current opinion that other phosphaturic hormones such as matrix extracellular phosphoglycoproteins, fibroblast growth factor 7, and secreted Frizzled-related Protein 4 may also be secreted in association with certain tumors, although their role in disease pathogenesis remains less clearly defined [1,4].

Although there is a lack of data on the real prevalence and incidence of the disease, it is estimated that TIO affects 0.40–0.10 per 100,000 individuals in the general population [5,6]. It seems to have a median age of 40–45 years at onset, and to affect both genders equally [7].

Pathophysiologically, FGF-23 excess disrupts mineral homeostasis by downregulating sodium–phosphate cotransporters (NaPi-2a/2c) in the proximal tubule and suppressing renal 1-α-hydroxylase activity. This dual mechanism leads to severe renal phosphate wasting and impaired vitamin D metabolism [8]. The resulting chronic hypophosphatemia severely compromises bone mineralization, leading to osteomalacia, which presents with a nonspecific constellation of symptoms—including diffuse pain, muscle weakness, and recurrent fractures. These symptoms frequently lead to misdiagnoses, as they are often attributed to rheumatological or musculoskeletal disorders [9], resulting in a significant diagnostic delay [4,10]. Consistent wth this concept, misdiagnosis at presentation is reported in 87.5% to 95% of cases [1].

In this setting, laboratory findings are crucial for a TIO diagnosis. They include: hypophosphatemia due to renal phosphate wasting, hyperphosphaturia and a low rate of tubular maximum phosphate reabsorption to glomerular filtration rate (TmP/GFR), as well as low or inappropriately normal 1,25(OH)_2_vitaminD_3_, while parathyroid hormone (PTH) and calcium concentrations are usually normal at least in the early stages of the disease [1,10]. However, the laboratory workup can be further complicated by “masking” factors. The profound 1,25(OH)_2_vitaminD_3_ deficiency induced by FGF-23 often triggers secondary hyperparathyroidism, which can progress to autonomous tertiary hyperparathyroidism over time [10]. When hypercalcemia and elevated PTH coexist, clinicians may prematurely conclude a diagnosis of primary hyperparathyroidism, leading to surgical interventions that normalize calcium but leave the underlying TIO untreated.

Here, we report a challenging case of TIO where a coexisting parathyroid disease initially masked the underlying phosphaturic disorder. This report highlights the importance of a systematic diagnostic workup in unresolved hypophosphatemia, particularly after parathyroidectomy. To contextualize this case, we performed a review of the literature and a search in PubMed of other patients with TIO and tertiary hyperparathyroidism. For this purpose, we used the combined terms “TIO”, hypercalcemic hyperparathyroidism”, “primary hyperparathyroidism”, and “tertiary hyperparathyroidism”, and we selected only English-written articles, while we excluded a few reports in other languages and/or abstracts.

## 2. Case Report

A 74-year-old patient was referred to our Endocrinology Unit with a long-standing history (~30 years) of bone pain, recurrent fragility fractures, nephrolithiasis and progressive muscle weakness. Biochemical evaluation revealed hypercalcemia (11.1 mg/dL; r.r. 8.5–10.5), severe hypophosphatemia (1.8 mg/dL; r.r 2.5–4.5), and elevated PTH (298 pg/mL; r.r. < 73), with sufficient 25(OH)vitamin D levels (36 ng/mL), as shown in Table 1. A diagnosis of primary hyperparathyroidism was made. The neck ultrasound and ^99m^Tc-MIBI scintigraphy identified a hyperfunctioning lesion at the lower pole of the right thyroid lobe.

The patient underwent parathyroid surgery, starting with the minimally invasive excision of the enlarged parathyroid gland that was localized preoperatively in the right inferior parathyroid gland. However, intraoperative PTH (ioPTH) monitoring showed an insufficient decline, with levels dropping from 536.7 pg/mL to only 413 pg/mL after 10 min from the excision of the right lesion. This necessitated a conversion to a bilateral neck exploration, which revealed a second pathological gland on the left side. Following its resection, ioPTH levels successfully dropped to 104.9 pg/mL after 10 min (>50% decrease from the highest pre-excision value [11]), indicating the complete removal of all hyperfunctioning parathyroid tissue, based on the Miami criteria [11]. Histopathology revealed an unusual combination: a parathyroid carcinoma on the right side and a parathyroid adenoma on the left. The lesion that was diagnosed as a carcinoma exhibited a solid trabecular pattern composed of round cells with finely granular cytoplasm (TTF1−, PTH+). The neoplasm was surrounded by a thick fibrous capsule with multiple foci of capsular infiltration and extracapsular extension into surrounding tissues. Notably, vascular invasion was identified, as evidenced by intravascular neoplastic emboli staining positive for PTH and CD31. The Ki67 proliferative index was 5%, while mitoses and necrosis were absent. To ensure oncologic radicality, after discussion in our multidisciplinary tumor board, a total thyroidectomy was subsequently performed. Total thyroidectomy rather than right lobectomy was chosen due to the presence of chronic lymphocytic thyroiditis and a left thyroid nodule. Genetic screening for multiple endocrine neoplasia (MEN)1 and MEN2A was negative.

During post-surgical follow-up, calcitriol (0.5 μg twice daily) and calcium carbonate (500 mg twice daily) were prescribed to support calcium homeostasis and bone remineralization in the early postoperative phase. While calcium and PTH levels normalized, as shown in Table 1, severe hypophosphatemia persisted (1.8 mg/dL) together with asthenia and bone pain. The persistence of hypophosphatemia prompted further metabolic evaluation, showing inappropriate phosphaturia (638 mg/24 h) and a significantly reduced TmP/GFR (1.15 mg/dL; r.r. 2.6–3.8). The presence of renal phosphate wasting with normal PTH levels effectively excluded recurrent hyperparathyroidism, pointing towards an FGF-23-mediated process. At this stage, oral potassium/sodium phosphate salts at the final dosage of five tablets daily were added to the therapy. Intact FGF-23 was found to be elevated (214 pg/mL; r.r. 23.5–95.4), supporting a diagnosis of TIO, as shown in Table 1.

**Figure 1:**
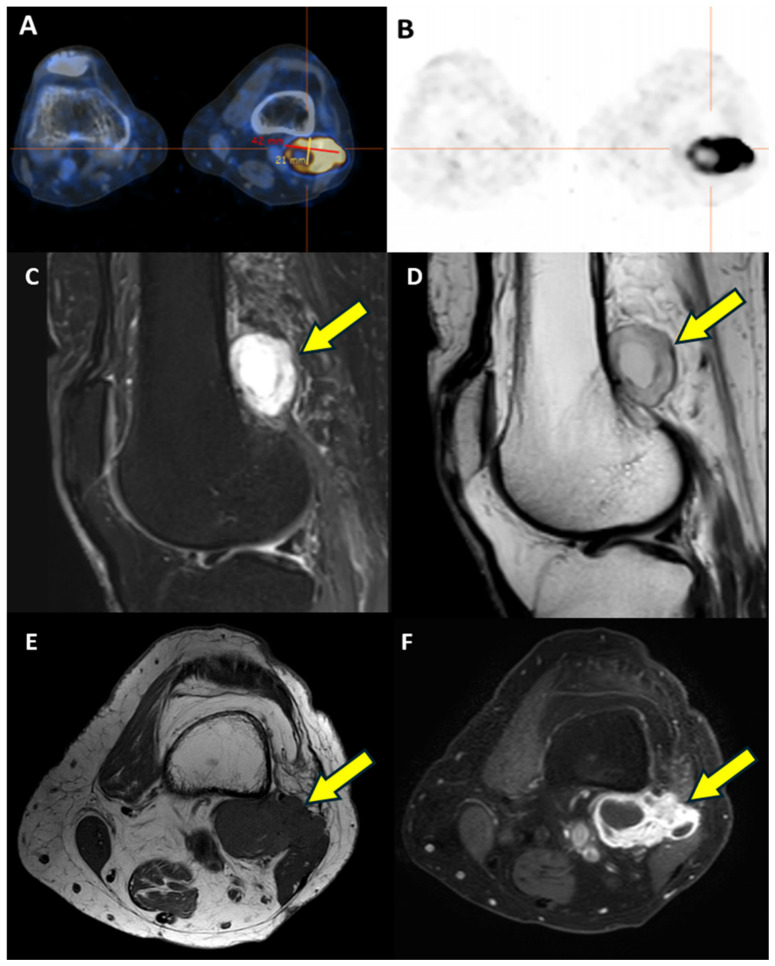
Imaging localization of the phosphaturic lesion. (**A**,**B**) ^68^Ga-DOTATOC PET/CT imaging showing intense focal radiopharmaceutical uptake in the left popliteal fossa. (**C**–**F**) MRI showed a well-defined multilobulated lesion (yellow arrow) with sharp margins, measuring 22 mm × 43 mm on the axial plane and 30 mm in longitudinal extension, within the soft tissues of the left popliteal fossa, slightly above the left lateral femoral condyle. The lesion appeared heterogeneously hyperintense on sagittal STIR (**C**) and T2-weighted TSE sequences (**D**) and hypointense on axial T1-weighted TSE images (**E**). After gadolinium-based contrast administration, T1-weighted TSE fat-sat images (**F**) showed intense peripheral and septal enhancement, with a multiloculated appearance and a non-enhancing core. The lesion was adjacent to the femoral cortex, popliteal neurovascular structures, and biceps femoris, without evidence of infiltration or regional lymphadenopathy.

A subsequent ^68^Ga-DOTATOC PET/CT localized a hypermetabolic lesion (42 mm × 21 mm × 41 mm; SUV max 7.2) in the left popliteal fossa (Figure 1). The magnetic resonance imaging (MRI) confirmed a multiloculated lesion, measuring 22 mm × 43 mm × 30 mm, consisting of a solid peripheral rim and a multichambered gelatinous core in contact with the lateral femoral cortex (Figure 1).

**Figure 2:**
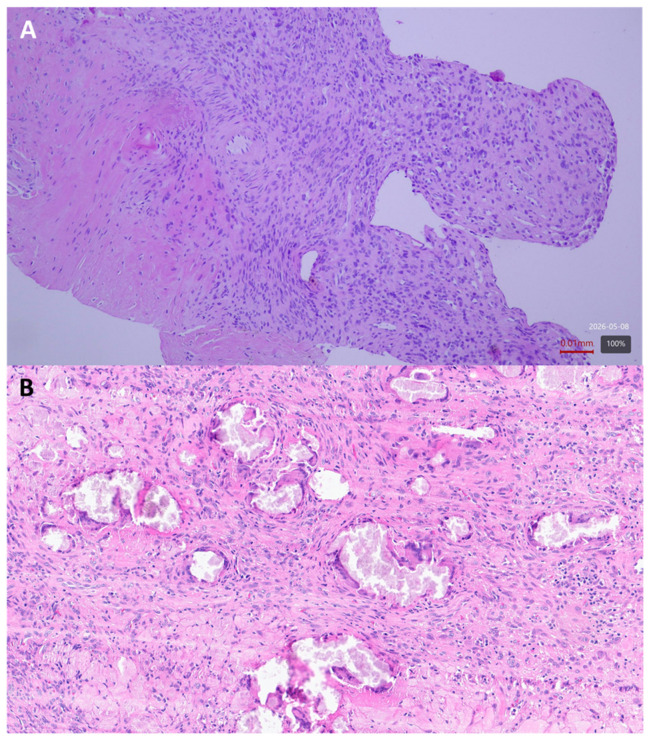
Histopathology of the popliteal fossa mass. (**A**) Microphotograph of the needle biopsy performed on the popliteal mass showing the proliferation of mononuclear histiocytoid elements with intervening multinucleated giant cells, reactive-appearing vascular structures, and a dense sclerotic matrix in a picture initially interpreted as a tenosynovial giant cell tumor. (**B**) Microphotograph of the surgical specimen demonstrating the presence of calcified needle-like material associated with a giant cell granulomatous reaction. Hematoxylin and eosin, original magnification ×10 (**A**) and ×20 (**B**).

To complete the preoperative workup, the patient underwent a core needle biopsy of the mass. Histopathological analysis showed a proliferation of round cells with slightly polymorphic nuclei and multinucleated giant cells, embedded in a sclerotic and grungy calcified matrix. No mitotic figures were observed. Immunohistochemistry was performed to assess the nature of the lesion, demonstrating negativity for broad-spectrum cytokeratins (AE1/AE3 and CAM5.2), PTH, GATA3, TTF1, CD163, chromogranin, synaptophysin, SOX10, CD34, HMB-45, and ERG (the latter being expressed only in vascular endothelia). A low proliferation index was confirmed by Ki67 staining, while CD56 showed granular cytoplasmic positivity with variable intensity (Figure 2).

Overall, the combination of clinical, biochemical, imaging, and histological data suggested the presence of a phosphaturic mesenchymal tumor with giant cell tumor-like features causing TIO. After discussion in a multidisciplinary tumor board, the patient underwent a complete surgical resection of the lesion through a posterior approach to the knee. Intraoperatively, a well-circumscribed multiloculated soft-tissue mass with a gelatinous component was identified in the popliteal fossa, in close relationship with the distal posterior femoral cortex, without macroscopic invasion of the surrounding neurovascular structures. The mass was removed by marginal *en bloc* excision, achieving a complete macroscopic resection, with microscopically negative margins confirmed on histopathological examination. Histopathological evaluation revealed a complex lesion characterized by fibrosis and giant-cell granulomatous inflammation associated with multifocal deposits of amorphous, needle-like calcified material. Overall, the morphological features were consistent with a tophaceous-like giant-cell granulomatous reaction. Of note, FGF-23 immunohistochemistry was not performed on either the core needle biopsy or the final surgical specimen, as the anti-FGF-23 antibody is not routinely available in our pathology department. Nevertheless, postoperatively, intact FGF-23 levels normalized immediately, leading to a progressive rise in serum phosphate (2.6 mg/dL); consequently, oral potassium/sodium phosphate supplementation was withdrawn. The patient reported the resolution of bone pain and a rapid recovery of motor function. Having said that, the final diagnosis of TIO was established through a multidisciplinary correlation of clinical, biochemical, imaging, and pathological findings.

## 3. Discussion

This case highlights how challenging the management of TIO can be, as in our case it was masked by hypercalcemic hyperparathyroidism due to a parathyroid carcinoma that delayed its diagnosis.

TIO is notoriously difficult to diagnose; the literature reports a median diagnostic delay ranging from 2.9 years in specialized centers [10] up to 28 years [12]. This has been put down to several factors: (1) clinicians might not be familiar with this rare paraneoplastic syndrome; (2) clinical presentation is nonspecific and often misinterpreted; (3) tumors are frequently very small and difficult to locate; and (4) biochemical features such as the elevation of calcium and PTH are misinterpreted as primary hyperparathyroidism [13]. Our case highlights that parathyroid disorders (hypercalcemic hyperparathyroidism) can coexist with TIO, leading to a misdiagnosis of primary hyperparathyroidism. Clinicians must maintain a high index of suspicion for TIO if hypophosphatemia persists after parathyroidectomy.

The underlying mechanism of hypercalcemic hyperparathyroidism in patients with TIO involves FGF-23-mediated inhibition of 1,25(OH)_2_vitaminD3 synthesis, which reduces intestinal calcium absorption and triggers compensatory PTH secretion. Over time, this chronic stimulation may lead to parathyroid autonomy. In addition, exogenous phosphate supplementation also stimulates parathyroid activity through sequestration of calcium [13].

The association between TIO and hypercalcemic hyperparathyroidism is gaining increasing attention. Recently, Ni et al. evaluated the prevalence, clinical characteristics and risk factors for hyperparathyroidism in a cohort of 91 patients with TIO [14]. They found that hyperparathyroidism was present in 45% of the patients with TIO, and that 41.6% had secondary hyperparathyroidism while 3.5% had tertiary hyperparathyroidism. Of note, patients with tertiary hyperparathyroidism had the longest disease duration and a higher rate of phosphate and calcitriol supplementation.

Table 2 summarizes the existing case reports of patients presenting with hypercalcemic hyperparathyroidism associated with TIO. Our systematic review of the literature identified 27 cases. The median age at diagnosis was 46 years (range 13–68). The median disease duration before recognizing the metabolic complication was 8 years (range: 3–38). In the majority of cases (76.9%, 20/26), hyperparathyroidism was identified after the onset of TIO symptoms. Regarding histopathology, benign lesions predominated, with adenoma (40.7%) and hyperplasia (33.3%) being the most frequent findings. Remarkably, no cases of parathyroid carcinoma were previously documented in this specific clinical setting, distinguishing our report as the first to describe such a rare malignancy in a patient with TIO-induced tertiary hyperparathyroidism. Consistent with this, our case is a reminder that parathyroid surgery serves not only as a therapy but also as a fundamental diagnostic tool.

Furthermore, in our case, histopathological analysis of the popliteal mass revealed a complex lesion characterized by a prominent giant-cell granulomatous reaction. This morphologic appearance validates Prader’s landmark early observation that such giant-cell-rich lesions can indeed be the source of FGF-23-mediated phosphaturia [34]. Historically, TIO was described in 1947 by Robert McCance [35], who reported the case of a patient with pain and weakness associated with low phosphate levels that improved only after a tumor found in the femur was resected [35]. However, the link between tumor and osteomalacia was established in 1959 by Prader, who postulated the production of a rachitogenic substance by a giant cell reparative granuloma of bone, given that the resection of the tumor led to the cure of the rickets [34]. The discovery of FGF-23 later provided the molecular basis for TIO, which is now recognized as a paraneoplastic syndrome caused, in most cases, by small phosphaturic mesenchymal tumors (PMTs).

PMTs most commonly arise in the soft tissues of the extremities and in the appendicular skeleton, although craniofacial bones and paranasal sinuses may also be involved. Histologically, they are typically composed of bland round-to-spindle cells, which are responsible for FGF-23 production, embedded in a variably vascular and extracellular matrix-rich stroma, often with characteristic “grungy” calcifications. Additional features may include multinucleated giant cells, fibrohistiocytic areas, myxoid matrix, woven bone formation, and, less frequently, adipocytic components. However, the relative proportions of neoplastic cells, vessels, matrix, calcifications, and giant cells vary considerably from case to case, making the diagnosis challenging, particularly in soft-tissue lesions. Indeed, a prominent giant-cell-rich or granulomatous reaction can heavily obscure the underlying neoplasm, leading to frequent diagnostic pitfalls. For instance, the most common misdiagnoses for PMTs include hemangiopericytoma, hemangioma, osteosarcoma, ossifying fibroma, giant cell tumor of bone, osteoblastoma, or purely reactive granulomas [2,4].

To overcome the limited diagnostic specificity of immunohistochemistry, combined immunoreactivity for FGF-23 and somatostatin receptors (SSTR2A), the latter being highly expressed in most PMTs [36], may increase diagnostic confidence [37]. Notably, SSTR2 expression might also be detected with somatostatin receptor-based imaging [38]. Somatostatin receptor-based imaging remains the gold standard for TIO localization, providing also crucial diagnostic support especially when the prominent giant-cell component or granulomatous-like features of the lesion might otherwise obscure its underlying neoplastic, phosphaturic nature [37,38]. In addition, it has been recently demonstrated that ^68^Ga-DOTATATE PET/CT has superior diagnostic accuracy compared to ^18^F-FDG PET/CT for the localization of TIO [38,39]. Consistent with this, in our patient, ^68^Ga-DOTATOC PET/CT was instrumental in accurately localizing the potential phosphaturic tumor.

Regarding their clinical behavior, PMTs exhibit a broad and often unpredictable biological spectrum [40] and complete excision remains the treatment of choice [41,42]. While the majority are benign and slow-growing, they can range from indolent lesions to locally aggressive tumors and, rarely (<10%), malignant forms capable of recurrence or distant metastasis [43]. According to Folpe et al. [40], histologically malignant PMTs are characterized by focal areas of high cellularity, high nuclear grade, and mitotic activity of >5/10 HPF, a high Ki-67 proliferation index and p53 expression, resembling an undifferentiated spindle cell sarcoma. In our case, the lesion displayed a favorable risk profile, lacking any of these features. This benign nature was further supported by the intraoperative findings of a well-circumscribed mass and the subsequent histological confirmation of microscopically negative resection margins.

Long-term surveillance is recommended [42,44], although no standard protocols have been universally established. In this clinical context, we implemented a follow-up strategy combining regular biochemical monitoring with local radiological reassessment via MRI starting 6 months after surgery. Clinically, fasting serum phosphate levels should be measured initially every 3–6 months and annually thereafter [10]. From an imaging perspective, an MRI of the surgical site is indicated 6 months postoperatively; subsequent MRI scans or ^68^Ga-DOTATOC PET/CT may be considered in the event of recurrent hypophosphatemia, rising FGF-23 levels, or in the presence of equivocal radiological findings. Nevertheless, given the broad biological spectrum of the disease, follow-up strategies must always be tailored to the individual patient’s presentation.

## 4. Conclusions

TIO is an extremely challenging condition. This case sheds light on the fact that hypercalcemic parathyroid disorders can coexist with TIO, and they may delay its diagnosis. Early integration of a meticulous biochemical follow-up and functional imaging is essential to prevent diagnostic delays and ensure the timely surgical resolution of this debilitating condition.

## Figures and Tables

**Table 1:** Laboratory tests.

Test	Presentation	2 Months After PTX	TIO Diagnosis	2 Months After Surgical Resection	6 Months After Surgical Resection
PTH (pg/mL)	298	64	49	58	54
S-Calcium (mg/dL)	11.1	9	9.6	10	10.3
S-Phosphate (mg/dL)	1.8	1.8	1.73	2.6	2.9
S-Creatinine (mg/dL)	1.34	1.14	1.18	0.99	0.99
25(OH)vitD (ng/mL)	36	26	38	27	30
FGF-23 (pg/mL)	/	/	214	93	58

PTX, parathyroidectomy; TIO, tumor-induced osteomalacia; PTH, parathyroid hormone; 25OHvitD, 25(OH)vitamin D; FGF-23, fibroblast growth factor-23.

**Table 2:** Association between TIO and hypercalcemic (tertiary) hyperparathyroidism.

Ref	Patient	Primary Tumor	Tumor Location	FGF-23	Disease Duration Before THPT Diagnosis	PTH	Pathology
Olefsky, 1972 [15]	40, M	Ossifying mesenchymal tumor	Pharynx	ND	12 y (A)	ND	Adenoma
Wyman, 1977 [16]	44, M	Osteolytic tumor	Tibia	ND	5 y (A)	ND	Transient hypercalcemia
Pollack, 1973 [17]	9, M	Non-ossifying fibroma	Radius	ND	4 y (A)	ND	Transient hypercalcemia
Firth, 1985 [18]	44, M	Chondrosarcoma	Femur + lung	ND	18 y (A)	540 *	Hyperplasia
Reid, 1987 [19]	57, F	PMT	Ankle	ND	16 y (B)	45 *	Hyperplasia
Heylen, 1999 [20]	52, F	Non-ossifying fibroma	Radius	ND	5 y (A)	137	Adenoma
Huang, 2000 [13]	39, F	Osteosarcoma	Scapula + lung	ND	7 y (A)	500	Adenoma
Sato, 2001 [21]	38, M	Chondroblastoma	Femur	ND	14 y (B)	246	Hyperplasia
Tartaglia, 2006 [22]	65, F	PMT	Groin	ND	17 y (B)	-	Hyperplasia
Tournis, 2007 [23]	68, F	PMT	Arm	ND	38 y (A)	235	Not operated
Nawrot-Wawrzyniak, 2009 [24]	54, M	HPC	Scapula	3330	12 y (B)	470	Adenoma
Markou, 2011 [25]	44, F	PMT + Malherbe epithelioma	Thigh + humerus	1035	3 y (B)	284	Adenoma
Elfenbein, 2012 [26]	67, M	PMT	Thigh	2520	ND (A)	218	Adenoma
Bhadada, 2013 [27]	56, M	Not operated	Femur (?)	1722.5	8 y (A)	109	Hyperplasia
Hu, 2015 [28]	52, F	PMT	Forearm	-	20 y (A)	823	Not operated
Koikawa, 2020 [29]	53, F	PMT	Ethmoid sinus	2878 ^§^	6 y (A)	106	Adenoma
Kumar, 2020 [30]	13, M	PMT	10th rib	535	5 y (B)	846	Adenoma
Kilbane, 2021 [31]	52, F	PMT	Pectineus muscle	2520	5 y (A)	112	Not operated
Salim, 2021 [32]	49, M	PMT	Lung	1330	8 y (A)	274.4	Not operated
Brociek-Pilczynska, 2022 [33]	42, F	GPC	Nasal cavity	872	5 y (A)	150	Adenoma
Ni, 2023 [14]	~46	PMT	ND	~1789 ^§^	7 y (A)	217.3	Hyperplasia
Ni, 2023 [14]	~46	PMT	ND	~1789 ^§^	6.5 y (A)	617.7	Adenoma + Hyperplasia
Ni, 2023 [14]	~46	PMT	ND	~1789 ^§^	18 y (A)	664.1	Hyperplasia
Ni, 2023 [14]	~46	PMT	ND	~1789 ^§^	10 y (A)	387.8	Adenoma
Ni, 2023 [14]	~46	PMT	ND	~1789 ^§^	11 y (A)	191	Hyperplasia
Ni, 2023 [14]	~46	PMT	ND	~1789 ^§^	7 y (A)	714.3	Adenoma
Ni, 2023 [14]	~46	PMT	ND	~1789 ^§^	17 y (A)	197	Hyperplasia

FGF-23 is RU/mL except for (^§^) where it is expressed as pg/mL; GPC, glomangiopericytoma; HPC, hemangiopericytoma; ND, not determined; PMT, phosphaturic mesenchymal tumor; PTH is expressed as pg/mL except for (*), where it is expressed as uIeq/mL.

## Data Availability

Data are contained within the article.

## Abbreviations

The following abbreviations are used in this manuscript:

^18^F-FDG	Fluorine-18 fluorodeoxyglucose
^68^Ga	Gallium-68
^99^mTc-MIBI	Technetium-99m sestamibi
AE1/AE3	Multi-cytokeratin cocktail AE1/AE3
CAM5.2	Low molecular weight cytokeratin CAM5.2
CD31	Cluster of differentiation 31 (endothelial cell adhesion molecule)
CD34	Cluster of differentiation 34
CD56	Cluster of differentiation 56 (neural cell adhesion molecule)
CD163	Cluster of differentiation 163
ERG	ETS-related gene
FGF-23	Fibroblast growth factor 23
GATA3	GATA binding protein 3
HMB-45	Human melanoma black 45
HPF	High-power field
ioPTH	Intraoperative parathyroid hormone
Ki67	Ki-67 proliferation index
MEN 1	Multiple endocrine neoplasia type 1
MEN2A	Multiple endocrine neoplasia type 2A
MRI	Magnetic resonance imaging
NaPi	Sodium–phosphate cotransporter
PET/CT	Positron emission tomography/Computed tomography
PMT	Phosphaturic mesenchymal tumor
PTH	Parathyroid hormone
PTX	Parathyroidectomy
r.r.	Reference range
SOX10	SRY-box transcription factor 10
TIO	Tumor-induced osteomalacia
TmP/GFR	Tubular maximum reabsorption rate of phosphate to glomerular filtration rate
TTF1	Thyroid transcription factor 1
